# Challenges in Evaluating Clinical Governance Systems in Iran: A Qualitative Study

**DOI:** 10.5812/ircmj.13421

**Published:** 2014-04-05

**Authors:** Elaheh Hooshmand, Sogand Tourani, Hamid Ravaghi, Hossein Ebrahimipour

**Affiliations:** 1 Health Sciences Research Center, Department of Health and Management, School of Health, Mashhad University of Medical Sciences, Mashhad, IR Iran; 2 Hospital Management Research Center, Iran University of Medical Sciences, Tehran, IR Iran; 3 School of Health Management and Information Sciences, Iran University of Medical Sciences, Tehran, IR Iran

**Keywords:** Clinical Governance, Qualitative Research, Iran

## Abstract

**Background:**

In spite of the pivotal role of clinical governance in enhancing quality of services provided by hospitals across the country, a scientific framework with specific criteria for evaluating hospitals has not been developed so far.

**Objectives:**

This study was conducted with the aim to identify the challenges involved in evaluating systems of clinical governance in Iran.

**Materials and Methods:**

For the purposes of this qualitative study, 15 semi-structured interviews with experts in the field were conducted in 2011 and the data were analyzed using framework analysis method.

**Results:**

Five major challenges in evaluating clinical governance include managing human resources, improving clinical quality, managing development, organizing clinical governance, and providing patient-oriented healthcare system.

**Conclusions:**

Healthcare system in Iran requires a clinical governance program which has a patient-oriented approach in philosophy, operation, and effectiveness in order to meet the challenges ahead.

## 1. Background

The phrase “clinical governance” was first coined in the UK NHS and rapidly became an international framework for quality improvement through increased professional knowledge and healthcare professionals’ accountability for providing clinical excellence (1). In fact, the emergence of clinical governance was due to the concerns raised about the quality and safety of services, and subsequent public dissatisfaction with healthcare system (due to heightened expectations), rising healthcare costs, and increased medical errors that prompted the deeply felt necessity of a new qualitative approach to resolve these issues (2). Besides, providing quality in the healthcare sector has always been a subject of huge importance to policy makers and politicians alike (3, 4). There are many approaches for enhancing quality of services in the healthcare sector, most of which fail due to cultural differences, types of organizations, and organizational climate of healthcare institutions and industrial organizations. In order to support healthcare organizations and combat such influences, clinical governance appears to be beneficial, as this approach helps organizations to be structurally more flexible and react swiftly to changes (5, 6). In studies by Scally and Donaldson, and Pearce and Swage, lack of standards and quality issues in providing healthcare services have been identified as the reasons for the need for clinical governance as a qualitative approach. These issues have also been of concern to governments (7-9). Implementation of clinical governance for enhancing quality of healthcare has had vastly positive results in various countries (10-14). The main challenge in establishing clinical governance is for the policies to translate into implementable actions (15). In other words, it is vital to design a system to evaluate clinical governance and ensure the realization of proper infrastructures that are conducive to the goals of this system (16). In the healthcare systems in different countries, different models are used to evaluate clinical governance. These models evaluate level of success of healthcare organizations in implementing this system (17). In Iran too, with existing experiences in the Ministry of Health, and some universities of medical sciences, it is expected that with proper implementation, in accordance with the conditions of healthcare system in Iran, clinical governance system be able to solve some of the healthcare problems (18, 19). Accordingly, the Health Policy Council has investigated attempts and researches in recent years, and proposed a plan for clinical services governance with seven principles, four in a meeting dated October 30th, 2009 [the MOHME official letters No.45025, 92561, 106083, and 113205 (Persian)]. This approach was stressed in the Fifth Development Plan and in the Health Policy Council, and has a pivotal role in accreditation of hospitals across the country as hospitals have been evaluated according to this approach in the past two years (20).

## 2. Objectives

Investigations have revealed that, so far, there are no published scientific documentations in the country regarding the evaluation criteria for clinical governance establishment in hospitals. Therefore, this study was conducted aiming at identifying the challenges involved in evaluating systems of clinical governance in Iran, and thus help in effective establishment of clinical governance in Iran’s healthcare system.

## 3. Materials and Methods

1. In this qualitative study, semi-structured interviews were conducted with 15 participants who were selected by the inclusion criteria for this study are:
2. Having effective experience in clinical governance field (more than one year) which means job experience at the Ministry of health;
3. Having related academic background and participating in clinical governance committee (more than 6 months).

These included five experts with relevant qualifications and with at least one published paper on the subject, three hospital managers who were responsible for implementation of clinical governance system in their hospitals, four clinical governance hospital committee members, and two staff experts of the state universities health deputy. Tables 1 and 2 shows the characteristics of interviewers. The selection process was at first purposeful (expert selection), and completed in snowball manner (21). Saturation method has been used for sampling and since after 12 interviews the 13th, 14th and 15th did not have any extra information therefore the s study sample was limited to 15 experts. An introductory letter was sent for the prospect interviewees asking them to set an appointment for the interview. Ten interviews were conducted face to face, two were on the phone, and two were through e-mails with two subsequent complementary interviews. All interviews were recorded and transcribed by one researcher (E.H) to the point of data saturation, each taking 45 to 60 minutes on average.

**Table 1.** Frequency of Interviewers Based on Education and Sex ^a^

	MS	PhD	GP	Total
**Gender**				
Female	0	1 (50)	1 (50)	2
Male	1 (7.69)	9 (69.23)	3 (23.07)	13
**Total**	1 (6.66)	10 (66.66)	4 (26.66)	15

^a^Data are presented in NO. (%).

**Table 2.** Frequency of Interviewers Based on Work Experience and Age ^a^

	< 2 Years	2-5 Years	> 5 Years	Total
**Experience**				
25-35	3 (50)	3 (50)	0	6
35-45	1 (14.28)	4 (57.14)	2 (28.57)	7
> 45	0	1 (50)	1 (50)	2
**Total**	4 (26.66)	8 (53.33)	3 (20)	15

^a^Data are presented in NO. (%).

Interviews were conducted in accordance with interview question guide, extracted from international clinical governance evaluation samples in a semi-structured questionnaire format (15, 17, 22, 23), arranged to question opinions and beliefs of the interviewees about the subject. Form and content validity of questions were confirmed by 3 clinical governance experts. Framework analysis was used for data analysis, comprising 5 stages of familiarization, identifying a thematic framework,

indexing, charting, mapping and interpretation (24). In the familiarization stage, a form containing information about each participant and summary of interview content was prepared. To create the initial conceptual guideline, several sessions were held by researchers. Then, this conceptual framework was examined through repeated assessment of each interview in the familiarization stage. One of the researchers (E.H) coded each interview separately, making a list of these codes and their association with the conceptual framework extracted from these interviews. At this stage, each section containing common interview information was given one or two specific codes. These codes were scrutinized in group discussion meetings with other researchers, and altered if necessary. This process was repeated a number of times for each interview. Then, in the charting stage, opinions of interviewees about each criterion were compared. Where necessary, for better understanding of what was said in the interviews, the original interview was referred to and notes were added. Atlas Ti software was used in all the above stages. All interviewees verbally consented to take part, and financial compensation was provided for their cooperation. This research was approved by the Medical Ethic Committee of Tehran University of Medical Sciences (No.90/123) and required to follow these ethical points:

1. Informed consent of all interviewees;
2. Give complete information about the purpose of research to interviewees;
3. The comments of interviewees will remain confidential.

## 4. Results

By the end of the analysis, fivemain themes were identified including human resource management, improving clinical quality, managing development, organizing clinical governance, and providing patient-oriented healthcare system. These encompass 16 sub-categories (Table 3).

**Table 3.** The Required Categories and Subcategories in the Evaluation System of Clinical Governance ^a^

	Results
**Categories**	
**Human resource management**	
Performance evaluation	12 (80)
Training and development	15 (100)
Personnel motivation	11 (73.33)
**Improving clinical quality**	
Clinical audit	14 (93.33)
Clinical effectiveness	13 (86.66)
Risk management	15 (100)
**Developing management**	
Allocation of resources	9 (60)
Policy and strategy	10 (66.66)
External audit	7 (46.66)
Information systems	11 (73.33)
Research activities	8 (53.33)
**Organizing clinical governance**	
Clinical governance structure	12 (80)
Clinical governance pre-requisites	14 (93.33)
**Providing patient-oriented healthcare system**	
Management of patient’s non-medical needs	14 (93.33)
Complaints	15 (100)
Patient’s participation in treatment process	11 (73.33)

^a^Data are presented in NO. (%).

### 4.1. Main Category 1: Human Resource Management

#### 4.1.1. Performance Evaluation

In order to ensure evaluating performance of human resources is in accordance with principles of clinical governance, attention must be paid to evaluation indices and methods “staff performance evaluation indices must be determined in accordance with clinical governance principles” (p1). Additionally, performance evaluation feedback should be presented to the staff and results should be used for performance improvement purposes. "All relevant documentations must be available in the hospital" (p5). "These documents may be used in clinical governance related processes such as planning training courses" (p1).

#### 4.1.2. Training and Development

Training and development may be used as tools for familiarizing personnel with principles and philosophy of clinical governance and for facilitating its establishment. “Clinical governance and its principles as a guideline along with training must be available to all. Belief cannot be created without training” (p 1, 5, 7). “Staff must be briefed during training courses, and these courses must be written and documented, and documents must be available” (p 3, 9).

#### 4.1.3. Personnel Motivation

Motivation and encouragement of the personnel through responding to their needs and their involvement in the organization’s programs, provides satisfaction and ultimately improves performance and quality of healthcare services. “Personnel’s needs should be anticipated to increase satisfaction” (p 8). “There should be evidence of a welcoming atmosphere for staff ideas and opinions regarding clinical governance, and verification of staff satisfaction through regular interviews with a target group must be conducted and documented, and positive performances must be appreciated" (p 1, 10).

### 4.2. Main Category 2: Improving Clinical Quality

#### 4.2.1. Clinical Audit

In the process of auditing, the hospital should specify which area is most important in deciding about standards and auditing process first, and then use the results in practice. “Hospitals should identify their priorities, and define their standards accordingly” (p 6).

#### 4.2.2. Clinical Effectiveness

To ensure correct establishment and implementation of guidelines, it is important that they would be available in hospitals and their application be facilitated. “Scientific evidence, guidelines, protocols, and standards of services must be available in hospitals and should be posted in places easily visible by staff so that work can be performed according to them” (p 5).

#### 4.2.3. Risk Management

A system of risk reporting in hospital wards, and a system of learning from mistakes is vital in risk management, which should lead to reduced incidence of mistakes. “Hospitals should have a system of assessment of reported mistakes, and potential risks should be identified so that intervention can be accordingly defined” (p 11). “We must develop a culture of learning from mistakes, which can greatly reduce the number of hazards” (p 14). “According to the official statistics, do the steps taken in line with risk management in hospitals truly reduce the incidence of mistakes or not?” (p 2)

### 4.3. Main Category 3: Development Management

#### 4.3.1. Allocation of Resources

To succeed in implementation of clinical governance and improvement of quality of activities, appropriate allocation of resources must be ensured. “Documentations of allocation of resources to different sections in the hospital must be investigated, and thus, ascertain their expression in activities related to clinical governance, and their correct and effective implementation in line with clinical governance in practice” (p 5).

#### 4.3.2. Policy and Strategy

Compliance regarding strategic and operational programs of the organization, and level of agreement of these with clinical governance goals, play an important role in successful implementation of clinical governance. “According to the previously developed strategic plans, stages of establishment of clinical governance and its organizational structure must be already identified” (p7). “Operational plans must be examined to determine how they would link with clinical governance” (p 11).

#### 4.3.3. External Audit

External audit reports must be continual and inclusive of strengths and hospital improvement points. “External audit reports must include recommendations for improving performance, and the influence of previous reports on the organization’s performance must be tangible” (p 13). “This process must be maintained over regular intervals. The hospital should clarify and define indicators and standards needed, and turn the results into elements that would elevate quality of the system” (p 15).

#### 4.3.4. Information Systems

Correct functioning of information systems and their ability to meet the needs of the hospital is crucial. “Have the hospital information systems been defined according to indices that are to be monitored and evaluated or not? In other words, could the hospital extract the information and data that it requires, and do they produce management reports?” (p 7).

#### 4.3.5. Research Activities

Research must be conducted into areas that would meet the needs of the hospital, and cover shortages. Also, results must be made use of. “Hospital shortages must be identified, and the areas where research is required declared to the universities and scientific institutions” (p 3). “Research results must be used in design of guidelines and processes. Results must also be shared with relevant organizations and foundations” (p 12).

### 4.4. Main Category 4: Organizing Clinical Governance

#### 4.4.1. Clinical Governance Structure

The main issue here is the presence of a clinical governance system and a person responsible for it. “Firstly, does a clinical governance system exist in the hospital? If so, is there a person responsible for it?” (p1) “Notification of various committees and examining their documentations and notification of persons responsible for clinical governance could reveal whether such structure truly exists in the hospital or not” (p 6).

#### 4.4.2. Clinical Governance Pre-requisites

In assessing these pre-requisites, as in any change in an organization, commitment of the senior management and cooperation of personnel are extremely important. “Regarding the clinical governance, information must reach all those involved in the processes. This could be achieved through training courses, where personnel are introduced to the philosophy and aims of clinical governance, and are made aware of information in these courses” (p 12).

### 4.5. Main Category 5: Patient-oriented Health Services

#### 4.5.1. Management of Patient’s Non-medical Needs

Assessment of level of hospital’s responsiveness to the community in providing care has been the main issue of concern to the participants in this study. “A responsive system is one that gives the patient a choice of services, and provides the service appropriate for the patient’s disease in shortest possible time” (p 5).

#### 4.5.2. Complaints

Evaluation of complaint management and the presence of a system for receiving complaints in hospital are highly important for management of patients’ needs and improving performance. “Hospital must have a system of receiving and analyzing complaints. In addition, a feedback system for the clients must also be in place, where the outcome can be seen in practice” (p 13).

#### 4.5.3. Patient’s Participation in Treatment Process

In this part, training the patient to choose type of the treatment is of special importance. “We must make sure by direct visualization that educational brochures and pamphlets are available in different wards” (p 12). “Decision about treatment of patients in the hospital must involve presentation of adequate information in a simple language to the patient and/or his relatives, and encourage patient’s participation in decision making process” (p 7).

## 5. Discussion

This qualitative study aimed to identify challenges involved in evaluating clinical governance in Iran. The need to determine performance indices based on principles of clinical governance, and using performance evaluation results in practice, is the main challenge faced by the healthcare system in Iran. Results of similar studies also indicate that principles of clinical governance must play a vital role in the process of staff evaluation and performance revision. In addition, the process of identifying educational and developmental needs in an organization must closely relate to performance evaluation process and its results (6, 15, 25, 26).

The main challenges identified and emphasized in education and development is taking advantage of education as tools for familiarization of employees with clinical governance and facilitating commitment in them. Studies show that improving quality that is required by clinical governance can only be achieved through continuous clinical staff training and learning (27). For successful implementation of clinical governance, the training goals must be based on the organization’s training requirements, the community it covers, and its personnel, and practical experiences must be used in its planning (1, 28-30). In the area of personnel motivation, the main challenge was use of appropriate motivational methods to create staff satisfaction and gain their cooperation to meet clinical governance goals. Relevant studies also emphasize necessity of staff participation to ensure the effectiveness of clinical governance process (31-33), as this system must be accepted by all employees and the whole organization must be committed to it (25, 32).

The need to identify audit priorities and application of its results in planning interventions are the main challenges in clinical audit. Taylor also emphasizes application of audit results in improving quality of services and believes that this process as well as being complementary, provides an evaluation based on evidence of the clinical governance cycle in the hospital (34). An audit must be indicative of improvement areas, and be conducted with participation of all employees (35). In the area of clinical effectiveness, applying scientific evidence and guidelines in hospitals were the main challenges. Relevant studies also have emphasized this point, and consider that the best decisions for patient services must be taken in accordance with these guidelines (36, 37). The presence of mistake-reporting systems in hospitals and learning from mistakes were the main challenges in risk management. Similar studies also have emphasized the importance of learning from mistakes and communicating information that results from these mistakes (15). Regular meetings in connection with medical mistakes are also important in this regard (34, 38). Allocation of resources in accordance with the principles and goals of clinical governance is another main challenge. Research confirms that clinical governance will only be able to change performance if extra resources are allocated for this purpose, or resources are reallocated to areas where change is most needed, and prioritized accordingly (5, 39, 40).

The main challenge regarding policy and strategy was compliance of strategic plans and organizational operations with the principles and goals of the clinical governance. Travaglia and Donoldson in their study have emphasized the need for concurrence of clinical governance programs with organizational goals (2, 41). Identifying organization’s strengths and weaknesses through external audit, and use of audit results in improving quality of the system was another main challenge, and similar studies have also stressed this point (42, 43). In the area of information systems, the main challenge was design of a system based on the requirements of the hospital with capability to monitor and evaluate indices. Scott and Gringer stress that performance indicators can be measured through information and audit systems (15, 44). The information about patients must be available to service providers, in a timely and confidential manner (45).

Planning research based on the needs of the hospital and application of results in designing guidelines are the main challenges in research activity area. Use of scientific research results in improving clinical decisions, publicising research information in and out of hospital, and determination of hospital’s research and development needs are elements also mentioned in relevant studies (15, 46). In the area of clinical governance structure, the main challenge is existence of clinical governance system and associated committees in the organization. The NHS and department of health in Australia emphasize the need for clinical governance managers with clear job description within the organization, and also the need for clinical governance committees in hospitals (47-49).

The commitment of senior managers and personnel participation in implementation of clinical governance are the main challenges in establishment of clinical governance. Accountability is the heart of clinical governance. Meaning that, not only should the healthcare professionals be trying to improve quality, they must show their commitment in practice, too (50). Senior managers’ commitment is crucially important in implementation of clinical governance (51). In the management of patient’s non-medical needs, hospital’s response to the community it covers is the main challenge. Runciman, Chandraharan, and Colla regard clinical governance as public response to services provided, and emphasize providing modern patient-oriented services, and guaranteeing improved clinical conditions (52-54). The need for active complaint follow-up and process of dealing with complaints are the main challenges in complaint management. Complaints must be effectively followed up by a complaint committee, and patients must receive feedbacks. This leads to improvement of processes in the hospital (1, 55-57). As for patient’s participation in the process of treatment, the need for patient education and adequate information in order to involve the patient in treatment process is another main challenge (58, 59).

To meet the existing challenges, healthcare system in Iran requires a program that includes the followings:

### 5.1. Philosophical and Ethical Basis of Clinical Governance

Creating positive values and attitudes about safety and quality through reinforcing accountability, continual improvement of services, increased capabilities of clinical staff, assurance of quality and focus on ethics.

### 5.2. Executive Basis of Clinical Governance

Planning and organizing governance for providing safety and quality through operational management, allocation of resources, strategic planning, risk management, accountability, reporting and managing crisis, and application of standards.

### 5.3. Effectiveness Basis of Clinical Governance

Improving the process of information exchange between medical staff, clinical efficiency, and use of evidence-based medicine, auditing, efficient Knowledge management, research, and use of clinical indicators.

### 5.4. Patient-oriented Approaches

Encouragement of patient’s participation in the process of treatment, supporting culture of re-organization, and focus on patient’s safety, patient’s satisfaction, and effective dealing with complaints. Our study has some limitations. Inclusion criteria for interviewees were used, and a snowball system was employed to find interviewees so some experts may have been missed. Our findings may have been different if the participants or researchers were changed. But the strong points of this study are: First semi-structured interviews with the specialist shows existing challenges in evaluating clinical governance system in Iran. Second, it Gives some recommendations to address those obstacles based on literature review.
